# Investigations into the involvement of NMDA mechanisms in recognition memory^☆^

**DOI:** 10.1016/j.neuropharm.2013.04.013

**Published:** 2013-11

**Authors:** E. Clea Warburton, Gareth R.I. Barker, Malcom W. Brown

**Affiliations:** School of Physiology and Pharmacology, MRC Centre for Synapric Plasticity, University of Bristol, Bristol BS1 3NY, United Kingdom

**Keywords:** Recognition memory, Perirhinal cortex, Prefrontal cortex, Hippocampus NMDA, Plasticity

## Abstract

This review will focus on evidence showing that NMDA receptor neurotransmission is critical for synaptic plasticity processes within brain regions known to be necessary for the formation of object recognition memories. The aim will be to provide evidence concerning NMDA mechanisms related to recognition memory processes and show that recognition memory for objects, places or associations between objects and places depends on NMDA neurotransmission within the perirhinal cortex, temporal association cortex medial prefrontal cortex and hippocampus. Administration of the NMDA antagonist AP5, selectively into each of these brain regions has revealed that the extent of the involvement NMDA receptors appears dependent on the type of information required to solve the recognition memory task; thus NMDA receptors in the perirhinal cortex are crucial for the encoding of long-term recognition memory for objects, and object-in-place associations, but not for short-term recognition memory or for retrieval. In contrast the hippocampus and medial prefrontal cortex are required for both long-term and short-term recognition memory for places or associations between objects and places, or for recognition memory tasks that have a temporal component. Such studies have therefore confirmed that the multiple brain regions make distinct contributions to recognition memory but in addition that more than one synaptic plasticity process must be involved.

This article is part of the Special Issue entitled ‘Glutamate Receptor-Dependent Synaptic Plasticity’.

## Highlights

• NMDAR blockade in PRH, mPFC and HPC produces different patterns of memory deficits.
• NMDARs within these brain regions make distinct contributions to recognition memory.
• NMDARs are also critical for synaptic plasticity in the same brain regions.
• More than one synaptic plasticity process must be involved in recognition memory.

## 1 Introduction

The judgement of prior occurrence has multiple potential component aspects involving, for example, different modalities, individual items and associations, objects, places and scenes, familiarity, recency and recollection. This review will concern what is known of the involvement of NMDA receptors in judgement of prior occurrence, recognition memory, for objects, places and associations between object and places in rats. Thus information concerning NMDA mechanisms related to recognition memory processes will be the focus of this review. The first part of the review will focus upon mechanisms of synaptic plasticity in those brain regions we know to be critical for recognition memory, notably the perirhinal cortex, hippocampus, and medial prefrontal cortex (mPFC) and the second part of the review will focus on behavioural evidence of the critical role of NMDA neurotransmission, from genetic studies, but more specifically from pharmacological manipulations of NMDA receptors, within these brain regions in the formation of recognition memory.

## 2 Plasticity mechanisms

Memory requires there to be changes in neuronal connectivity that are maintained across time. The leading hypothesis is that such changes involve synaptic plasticity. The involvement of NMDA receptors in synaptic plasticity has been widely investigated ever since the seminal paper by Collingridge et al. (1983), Herron et al. (1986). The selective antagonist, AP5, of the NMDA receptor allows common mechanisms for inducing plasticity to be targeted without affecting normal low-frequency synaptic transmission (though high frequency transmission may be affected) (Bliss and Collingridge, 1993). Thus NMDA receptor activation has been shown to be necessary for the most common (though not all) forms of long-term potentiation (LTP) and long-term depression (LTD) in the hippocampus (Bashir and Collingridge, 1992; Malenka and Nicoll, 1993). Importantly, NMDA receptors are involved in the induction rather than maintenance of such plasticity (Collingridge et al., 1983). The details of NMDA receptor-dependent plasticity induction mechanisms are beyond the scope of this review. Moreover, reported effects will be restricted to those applicable to adult rather than immature cortex; the plasticity mechanisms are correspondingly more easily related to mnemonic rather than developmental processes. It should be noted that most detailed studies of synaptic plasticity have used brain slices and that the precise conditions within local networks during plasticity induction are not necessarily exactly those pertaining during memory formation in the intact brain. In particular, experimental induction of LTD requires stimulation with low frequency electrical pulses over many seconds while, at least in perirhinal cortex, reductions in neuronal responsiveness can be produced rapidly, in even <1 s (Brown and Xiang, 1998; Fahy et al., 1993; Miller et al., 1993). Moreover, importantly, NMDA receptor antagonism may have effects relating to the summation and synchronisation of action potentials in addition to blocking the induction of common forms of LTP and LTD. Accordingly, AP5 (and other NMDA receptor antagonists) may have effects on information processing and transmission as well as plasticity (Daw et al., 1993; Schiller and Schiller, 2001; Larkum and Nevian, 2008; Augustinaite and Heggelund, 2007; Hunt and Castillo, 2012): the behavioural effects (including amnesia) of blocking NMDA receptors, cannot therefore be attributed with certainty to blocking LTP and LTD. With these caveats in mind there have been a number of studies which have provided evidence that LTP and LTD-like mechanisms mediate the formation of distinct learning and memory processes including fear conditioning (Whitlock et al., 2006) and memory for object-location configurations (Kemp and Manahan-Vaughan, 2012; Goh and Manahan-Vaughan, 2013). Further weak synaptic plasticity has been shown to be strengthened by a concomitant learning event, suggesting that the same cellular mechanisms may underlie both synaptic plasticity and learning (Goh and Manahan-Vaughan, 2012).

What is known of the role of NMDA receptors in plasticity mechanisms in brain regions implicated in recognition memory processes will now be considered. There is strong evidence for the involvement of the perirhinal cortex, hippocampus, temporal association cortex and mPFC in aspects of recognition memory (Ennaceur et al., 1996; Mumby and Pinel, 1994; Bussey et al., 1999; Norman and Eacott, 2004; Barker et al., 2007; Barker and Warburton, 2011; Hannesson et al., 2004a; Ho et al., 2011). Other contributions in this volume review in detail the role of NMDA receptors in the hippocampus (references in this issue). Antagonism of NMDA receptors by AP5 blocks induction of both LTP and LTD in the adult perirhinal cortex (Bilkey, 1996; Banks et al., 2012; Cho et al., 2000; Griffiths et al., 2008; Ziakopoulos et al., 1999). However, the induction of LTD in adult perirhinal cortex maintained in vitro also involves mGlu receptor activation (Cho et al., 2000), so that differences have been established between basic plasticity mechanisms in hippocampus and perirhinal cortex. Presumably evolution would make possible the exploitation of such plasticity differences to effect different memory processes in different cortical structures.

Notably, in both hippocampus and perirhinal cortex, LTP and depotentiation (the reversal of previously induced LTP) are dependent on NMDA receptors containing GluN2A subunits, whereas LTD is dependent on NMDA receptors containing GluN2B subunits (Bartlett et al., 2007; Liu et al., 2004; Massey et al., 2004; Morishita et al., 2007). Thus antagonists that have selective actions on NMDA receptors containing GluN2A or GluN2B subunits may potentially be used to investigate the dependency of recognition memory on either LTP-like or LTD-like mechanisms.

The NMDA-receptor dependency of plasticity mechanisms has not been studied in temporal association cortex in the rat. In rat mPFC, however, both LTP and LTD have been demonstrated (Hirsch and Crepel, 1990, 1992; Izaki et al., 2003). Interestingly while LTP induction in the mPFC is NMDA receptor-dependent (Hirsch and Crepel, 1991; Huang et al., 2004; Jay et al., 1995; Vickery et al., 1997), only NMDA receptor-independent mechanisms of LTD have been found in this region (Banks et al., 2012; Caruana et al., 2011; Hirsch and Crepel, 1991; Huang and Hsu, 2010; Lafourcade et al., 2007).

## 3 Behavioural studies

Behavioural studies relating recognition memory processes to NMDA receptor mechanisms will now be reviewed. In the rat, recognition memory has been extensively studied by using the species' instinctive tendency to explore novelty. Such procedures based on preference for novelty have the advantage that differential association with reinforcement is avoided when novel and familiar situations are compared. The effects of NMDA receptor antagonism have been studied using four such recognition memory procedures – involving objects locations objects associated with particular places and temporal order. The procedures involve an acquisition or sample phase, a delay and a choice or test phase (for temporal order there are two or more sample phases and delays). In each of these procedures a rat familiarises itself with one or more objects and/or places during the acquisition phase through spontaneous exploration. At test, following a variable retention delay, exploration of what has been familiarised is compared with exploration of something newly introduced (Ennaceur and Delacour, 1988).

### 3.1 Object recognition memory

**Fig. 1:**
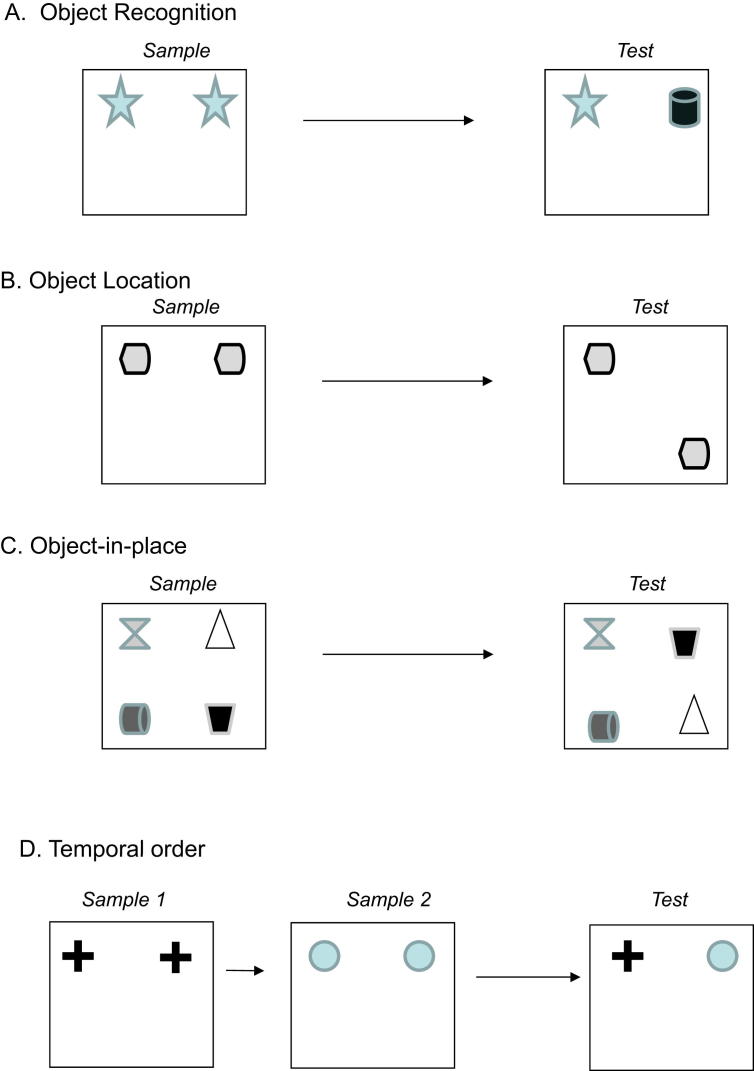
Diagram of the four object recognition memory tasks. A: Novel object recognition task. B: Object location task. C: Object-in-place task. D: Temporal order task.

In the standard object recognition memory task (OR) two objects are shown in the acquisition phase and during the test phase exploration of a familiar and a novel object is compared (see Fig. 1A). A number of studies now show that hippocampal or fornix lesions produce no effect in object recognition (Bussey et al., 2000; Mumby et al., 2002; Winters et al., 2004; Forwood et al., 2005; Good et al., 2007; Langston and Wood, 2010) although other studies have reported significant impairments (Clark et al., 2000, 2001). A recent study in our laboratory has established that both perirhinal cortex and the hippocampus are necessary for task solution if the two objects explored in the acquisition phase are different (G.R.I. Barker unpublished); however, only perirhinal cortex and not the hippocampus is required if the two objects explored at acquisition are identical copies of each other (Barker and Warburton, 2011; Winters et al., 2004). It is this latter (two rather than three object) version of the task that has been used, in the main, to study perirhinal NMDA receptor involvement in recognition memory.

Systemic and intracerebral administration of NMDA receptor antagonists have been shown to produced impairments in OR. Thus pre-training or post-training systemic administration of the non-competitive NMDA receptor antagonist MK801 impaired memory at 90 min and at 24 h suggesting that NMDA receptors are critical for both acquisition and consolidation (deLima et al., 2005). Similarly systemic administration of the competitive NMDA receptor antagonist (6)-3-(2-Carboxypiperazin-4-yl)-propanephosphonic acid (CPP) has been shown to block object familiarisation (Goh and Manahan-Vaughan, 2013) OR was also impaired when localised infusion of AP5 via cannulae placed bilaterally in perirhinal cortex was used to antagonise NMDA receptors during acquisition, with memory measured after a 3 h or 24 h delay (Barker et al., 2006; Winters and Bussey, 2005). However, the effect of AP5 on consolidation is equivocal as immediately post-acquisition intra-perirhinal infusion produced impairment in one study (Winters and Bussey, 2005) but not in another (Barker et al., 2006). Impairment has also been reported when AP5 was present during both consolidation and retrieval, the memory delay being 25 min (Abe et al., 2004); but when AP5 is present only during retrieval (i.e. before and during the choice phase) no effect is found (Barker et al., 2006; Winters and Bussey, 2005). If impairment is produced by post-acquisition infusion of AP5, the argument that NMDA receptor antagonism produces impairment by acting solely at acquisition is undermined and, correspondingly, the link between NMDA's roles in plasticity induction and memory are weakened.

Unexpectedly, AP5 infused into to perirhinal cortex to be active during acquisition does not impair recognition memory measured after a 5 min or a 20 min delay (Barker et al., 2006; Winters and Bussey, 2005) – though as mentioned above, Abe et al. (2004) found impairment at 25 min using a higher dose of AP5. Generally, it seems the memory delay must be ≥ 1 h before impairment is produced (Barker and Warburton, 2008). This time course of impairment conflicts with the prediction from studies of perirhinal plasticity mechanisms (Cho et al., 2000; Ziakopoulos et al., 1999) that AP5 should block plasticity induction, i.e. that impairment should occur at all delays. Rather than consequently rejecting the memory impairment as being a result of AP5's block of plasticity induction, the possibility must be considered that the effects of AP5 are being masked. Use of antagonists of other receptors (kainate or muscarinic cholinergic) establishes that perirhinal cortex is necessary for OR measured after short (≤1 h) delays (Barker et al., 2006; Tinsley et al., 2011), so an alternative, independent, masking memory is not being held outside perirhinal cortex. However, a second non-NMDA-dependent recognition memory mechanism has been found in perirhinal cortex, and this mechanism can support mnemonic behaviour at delays ≤1 h but not at longer delays (Barker et al., 2006; Tinsley et al., 2011). Hence there is a possible second memory mechanism that can mask the effects of AP5 at short delays. This finding means that AP5 may indeed produce recognition memory impairment by blocking perirhinal plasticity mechanisms, but it also implies that NMDA-dependent processes are not the only plasticity mechanisms supporting recognition memory within perirhinal cortex.

Studies in vitro (discussed briefly above) raised the possibility of selectively impairing either LTP or LTD mechanisms in the perirhinal cortex by using antagonists of GluN2A or GluN2B subunit-containing NMDA receptors respectively. It is predicted, from evidence obtained through in vivo electrophysiological studies, that a synaptic weakening mechanism should underlie recognition memory in the perirhinal cortex (Brown and Xiang, 1998). Indeed, interfering with synaptic weakening by selectively preventing the activity-dependent removal of AMPA receptors from the synaptic membrane (i.e. blocking the expression of plasticity rather than its induction) does impair OR at all memory delays (Griffiths et al., 2008). Further transgenic studies demonstrated that GluN2B overexpression in the forebrain (including the hippocampus and cortex) of both mice and rats significantly enhanced OR (Tang et al., 1999; Wang et al., 2009), although such studies cannot establish the precise neural substrate of this enhancement. Intra-perirhinal infusions of GluN2A or GluN2B antagonists, however produced an impairment of OR at a 24 h delay only when antagonism was both of receptors. Thus antagonists selectively targeting either GluN2A or GluN2B containing NMDA receptors on their own produced no impairment (Barker et al., 2006). This unexpected finding raises the possibility that there are compensatory mechanisms available to long-term recognition memory formation within perirhinal cortex during the induction of plasticity when either GluN2A or GluN2B receptors alone are inactivated. When both receptor subtypes were inactivated no such compensation was observed. Such compensatory mechanisms might involve different means of increasing the levels of intracellular calcium or convergence within other intracellular signalling pathways. Alternatively, there could be two independent mechanisms in the perirhinal cortex which underlie long-term object recognition memory, one dependent on a process used in LTP and another dependent on a process used in LTD, either being capable of supporting familiarity discrimination at long delays.

As stated previously a number of ablation studies indicate that hippocampus is not necessary for the successful performance of the two object version of the spontaneous OR task (Mumby et al., 2002; Winters et al., 2004; Forwood et al., 2005; Good et al., 2007; Langston and Wood, 2010; Barker and Warburton, 2011). However one study demonstrated that CA1-specific NMDA receptor 1 subunit-knockout mice impaired object recognition memory (Rampon et al., 2000) and another showed that intra-hippocampal infusion of AP5 before acquisition impaired the two object OR task after a delay of 3 h, though not of 5 min (Baker and Kim, 2002). Interestingly there is also a report that intra-mPFC infusion of AP5 after acquisition, in the three object OR task, impaired performance after a delay of 24 h, but not 3 h (Akirav and Maroun, 2006), although more recent lesion studies have demonstrated that the mPFC is not involved in familiarity discrimination (Barker et al., 2007**).**

In contrast, both lesions and local infusion of AP5 compromising temporal association cortex adjacent to perirhinal cortex impair OR (Ho et al., 2011). The impairment is seen at long (24 h) but not short (≤20 min) delays. Thus object recognition memory at long delays requires the unimpaired operation of this temporal association cortex as well as perirhinal cortex, including activity involving NMDA receptors in both regions. This temporal association cortex is not necessary at short delays. At short delays perirhinal cortex is necessary, though not its NMDA receptors.

In sum, in the two object version of OR, NMDA receptor neurotransmission in the perirhinal cortex and temporal association cortex during memory encoding is critical for the formation of long term >1 h, but not shorter-term memory. In the three object version of OR memory and for delays of at least 3 h, NMDA receptors appear to be involved in the hippocampus during acquisition and in the mPFC during consolidation.

### 3.2 Object location

In the standard object location recognition memory task (OL), a rat explores two identical objects in the acquisition phase. One of the objects is then moved to a new location and, after a delay, the rat explores the objects again (Fig. 1B). Typically the moved object is explored more than the one remaining in the same position. This task may be performed by remembering which spatial positions have or have not occupied previously.

Lesion studies have established that successful performance of the task requires the hippocampus (and fornix) but not perirhinal cortex or mPFC (Barker and Warburton, 2011; Bussey et al., 2000; Ennaceur et al., 1996). Intra-hippocampal infusions of AP5 before acquisition impair OL memory tested following a 1 h delay (Barker and Warburton, 2009). Thus NMDA receptor activation in the hippocampus is essential for recognition memory for familiar objects presented in a novel location. No experiments have been performed to establish if this memory impairment is produced by a loss of an LTP-like and/or LTD-like mechanism.

### 3.3 Object-in-place

Recognition memory for the association of objects with their positions – object-in-place (OiP) memory – may be measured as follows. In the acquisition phase a rat explores four different objects. The positions of two of the objects are then exchanged. At test after a delay, all four objects are the same and the same four positions are occupied; what is novel is the positioning of two of the objects (see Fig. 1C). If exploration of the two exchanged objects is greater than for the two unmoved objects, then the rat shows evidence of memory for the previous positions of the objects, i.e. a memory for the association of particular objects and particular places.

Ablation studies have established that the integrity of the hippocampus, perirhinal cortex and mPFC are all necessary for successful performance of the OiP task (Barker et al., 2007; Bachevalier and Nemanic, 2008; Wilson et al., 2008; Komorowski et al., 2009; Barker and Warburton, 2011). Moreover, interactions of each of these structures with the others are also necessary, i.e. the three structures are part of an interconnected neural circuit essential for the behaviour (Barker et al., 2007; Lee and Solivan, 2008; Barker and Warburton, 2011).

Bilateral infusion of AP5 into the hippocampus or mPFC so as to be active during acquisition impairs OiP memory following either a short (5 min) or a longer (1 h) retention delay (Barker and Warburton, 2008, 2009). There is no effect on retrieval of AP5 infusion into either structure. Hence in both structures the integration or association of object and place information over either a short or a long delay relies upon NMDA receptors during acquisition. The behavioural impairment is consistent with the expected consequences of AP5's interference with the induction of LTP and LTD-like plasticity mechanisms that depend upon NMDA receptor activation. As NMDA-dependent LTD mechanisms have yet to be found in the mPFC, it must currently be concluded that an impairment of LTP-like plasticity mechanisms is the probable cause of the memory deficit.

These pharmacological studies provide clear evidence supporting the link between synaptic plasticity mechanisms in the hippocampus and object-place learning, although the effects of AP5 on behaviour may be mediated through either LTP-like or LTD-like mechanisms. A recent study demonstrated that the presentation of novel object-place configurations can induce LTD in the rodent hippocampus through an NMDA receptor dependent mechanism (Kemp and Manahan-Vaughan, 2012; Goh and Manahan-Vaughan, 2012, 2013) providing evidence for a direct relationship between NMDA receptor dependent transmission, LTD and object-place learning in the hippocampus.

In parallel to the findings for the hippocampus and mPFC, infusion of AP5 bilaterally into perirhinal cortex so as to be active during acquisition impairs long-term OiP tested following a 1 h delay, and there is no effect on retrieval (Barker and Warburton, 2008). However, in marked contrast, AP5 infused into perirhinal cortex has no effect on acquisition of shorter-term (5 min delay) OiP (Barker and Warburton, 2008). This delay dependency of the amnesic action of AP5 echoes that found for OR (Barker et al., 2006; Winters and Bussey, 2005). Hence antagonism of perirhinal NMDA receptors produces the same pattern of delay-dependent acquisition impairment in the OiP task as in the OR task: impairment only at long (≥1 h) delays.

The necessity for concurrent NMDA receptor activation within the interconnected neural circuit involved in OiP has been explored by making crossed simultaneous unilateral infusions of AP5 into pairs of the three structures in the circuit. Unilateral infusions of AP5 into the hippocampus and mPFC in opposite hemispheres impair both shorter-term and long-term OiP (Barker and Warburton, 2009). In contrast, crossed unilateral infusions of AP5 into the perirhinal cortex and mPFC, or the perirhinal cortex and hippocampus, produce a significant impairment in long-term OiP memory, while shorter-term memory is unaffected (Barker and Warburton, 2009). Accordingly, a non-NMDA-dependent mechanism within perirhinal cortex is sufficient to sustain recognition memory for associations of objects and places at short delays, as also is found for non-associative object recognition memory.

The similarity in the time course of effects on memory of NMDA receptor antagonism in perirhinal cortex in the two tasks (object and object-in-place recognition memory) suggests that the same perirhinal mechanism is being affected. This suggests that perirhinal cortex provides a corresponding function for the two tasks. The most obvious function required by both tasks is registering that a particular object has been experienced. Thus it may be proposed that perirhinal cortex's contribution to object-in-place recognition memory is to store and then signal information about object occurrence. The plasticity mechanisms necessary to task solution in the hippocampus and mPFC must differ from those in the perirhinal cortex as NMDA antagonism in the former structures produces impairments in the OiP task at short as well as long delays. In particular, there can be no NMDA-independent short-term plasticity/memory mechanism in hippocampus and mPFC, capable of sustaining the behaviour in contrast to perirhinal cortex. The results of many studies indicate that the hippocampus processes information about spatial locations. Hence the contribution of mPFC to the circuit may be to associate hippocampally provided location information with perirhinally provided object information (Barker et al., 2007; Barker and Warburton, 2011).

### 3.4 Temporal order

A rat's memory for the order of the prior occurrence of objects may be investigated by showing two copies of an object in a first acquisition phase followed, after a delay, by two copies of a different object in a second acquisition phase. After a further delay the differential exploration of a copy of each of the objects may be tested (Fig. 1D). The expectation is that the object seen more recently will be less explored than that seen longer ago. In contrast, if a drug blocking acquisition is infused so as to be active during the second acquisition phase, greater exploration of the object seen at that time is to be expected, even though it was explored more recently. It should be noted that such a TO task may be solved on the basis of more than one type of information – for instance, relative familiarity, primacy, recency, the remembrance of temporal order. In addition when drug infusion is before the second acquisition phase, reconsolidation mechanisms for the object shown in phase one may be affected along with consolidation mechanisms for the object shown in phase two. The possibility that the TO task might be solved by forgetting the first object due to interference was ruled out in a recent study showing intact recognition memory (OR) for the objects presented in either sample phase one or two (Barker and Warburton, 2011).

Permanent lesions have established that TO recognition memory is dependent upon the same circuit of three structures as OiP memory, i.e. perirhinal cortex, mPFC and hippocampus (Barker et al., 2007; Barker and Warburton, 2011; Hannesson et al., 2004a,b; Mitchell and Laiacona, 1998). Also as for OiP, successful TO memory requires these regions to interact (Barker and Warburton, 2011; Barker et al., 2007; Hannesson et al., 2004a).

AP5 infused bilaterally into the mPFC before the second acquisition phase produces impairment of TO memory: no preference in exploration is found between the objects shown in the first and second acquisition phases. In contrast intra-perirhinal, infusion of AP5 produced an impairment such that the animals showed a preference for object seen in the second sample phase, suggesting different components of the trace were held in the two regions (Barker and Warburton, 2011). The delay between the two acquisition phases was 1 h and that from the second acquisition phase to the test was 3 h; shorter memory delays were not tested. Crossed unilateral infusion of AP5 into perirhinal cortex and mPFC significantly disrupted TO memory, indicating that the two structures needed to interact (Barker and Warburton, 2011). Thus for TO, as for OiP recognition memory, perirhinal and mPFC NMDA receptors are critical for acquisition when tested after a long delay. A possible additional role for NMDA receptors in reconsolidation in this task cannot be ruled out.

## 4 Conclusions

NMDA receptor neurotransmission clearly plays a critical role in the formation of object recognition memory in multiple brain regions. Within the perirhinal cortex and hippocampus NMDA receptors are known to be involved in both LTP and LTD. NMDA receptors within the perirhinal cortex and area TE are critical for the formation of long-term OR, but not shorter term OR, suggesting that a non-NMDA receptor dependent form of plasticity within these cortical regions must also be involved. NMDA receptors in the perirhinal cortex are also involved in TO and associative OiP recognition memory, with long but not short suggesting that the same NMDA-receptor dependent mechanism, within the perirhinal cortex, may underlie both associative and non-associative forms of recognition memory. In the mPFC and hippocampus, however, NMDA receptor neurotransmission is necessary for both short-term and long-term OiP memory and also for LTD in the hippocampus which has been shown to be directly linked to the formation of object-place learning. Accordingly in mPFC and hippocampus the NMDA-dependent mechanism can support shorter-term recognition memory. This finding establishes that different cortical regions do not all employ the same plasticity mechanisms to support recognition memory processes, although the nature of these differences needs more direct investigation.
